# The Clinical Application of Growth Hormone and Its Biological and Molecular Mechanisms in Assisted Reproduction

**DOI:** 10.3390/ijms231810768

**Published:** 2022-09-15

**Authors:** Peipei Pan, Xuefeng Huang

**Affiliations:** Department of Reproductive Medicine, The First Affiliated Hospital of Wenzhou Medical University, Wenzhou 325000, China

**Keywords:** growth hormone, insulin-like growth factor, steroidogenesis, primordial follicle activation, poor ovarian responder, assisted reproductive technology

## Abstract

Growth hormone (GH) has been used as a co-gonadotrophin in assisted reproduction, particularly in poor ovarian responders. The application of GH has been alleged to activate primordial follicles and improve oocyte quality, embryo quality, and steroidogenesis. However, the effects of GH on the live birth rate among women is controversial. Additionally, although the basic biological mechanisms that lead to the above clinical differences have been investigated, they are not yet well understood. The actions of GH are mediated by GH receptors (GHRs) or insulin-like growth factors (IGFs). GH regulates the vital signal transduction pathways that are involved in primordial follicular activation, steroidogenesis, and oocyte maturation. However, the therapeutic windows and duration of GH administration during assisted reproductive technology require further investigation. The review aimed to clarify the role of GH in human fertility from a molecular and biological point of view to provide evidence for proper GH administration.

## 1. Introduction

Growth hormone (GH) is a 191-amino acid that is generally considered to be secreted by adenohypophysis cells. Adjuvant treatment with GH in assisted reproductive technology (ART) plays a vital role in follicle development, steroidogenesis, oocyte maturation, ovulation, corpus luteum (CL) function, oocyte quality, and ovarian response to the administration of exogenous hormones [1,2,3,4]. GH reaches the target cell membranes, binds to the GH receptors (GHRs), triggers signaling inside cells, and then produces a series of physiological effects. The gene expression of GH is not confined to the pituitary gland, since its mRNA and the protein that is coded by it are produced in many extrapituitary sites, such as testes and ovaries [5,6]. Substantial studies have shown the presence of GH/GHRs in the ovaries of various organisms, including fish, chicken, rats, mice, horses, pigs, monkeys, and humans [7,8,9,10,11,12,13]. The occurrence of GHRs in ovaries indicates that these organs are a target site of GH action. GHRs are expressed in ovarian granulosa cells, theca cells, oocytes, cumulus cells, mammary glands, placentae, and the uterus [14]. In addition, GH and its receptors are expressed in all compartments of ovaries and change according to physiological conditions [15]. Since GH is involved in the regulation of reproductive functions, it has been used as a therapeutic option in ART for more than 30 years, especially in poor ovarian responders (PORs) [16,17,18,19,20,21], polycystic ovary syndrome (PCOS) [22,23], and poor embryonic development [24,25]. However, the effects of GH supplementation on the clinical outcomes, especially live birth rates (LBRs), for those undergoing ART remains controversial, and the underlying mechanisms are still not clear. Therefore, the aim of this review was to clarify the role of GH in human fertility, reveal the relevant mechanisms that are involved in GH actions, and provide evidence for proper GH administration.

The PubMed, Medline, and Google Scholar databases were searched for relevant papers in the published literature using combinations of the following search terms: “growth hormone”, “GH”, “insulin-like growth factor”, “IGF”, “poor ovarian responder”, “POR”, “assisted reproductive technology”, “ART”, “in vitro fertilization”, “IVF”, “live birth rate”, “pregnant outcome”, “intracytoplasmic sperm injection”, “ICSI”, “in vitro maturation” “IVM”, “polycystic ovarian syndrome”, “PCOS”, “ovary”, “oocyte”, “granulosa cell”, “theca cell”, “anti-Mullerian hormone”, “AMH”, “primordial follicle”, “nuclear”, “cytoplasm”, “poor embryonic development”, “signaling”, “pathway”, “randomized controlled trial”, “sensitivity”, “gonadotropin”, “FSH”, “steroidogenesis”, and “mitochondrial”.

## 2. The Application of Growth Hormone in Clinical Practice

Several studies have demonstrated that GH cotreatment with controlled ovarian stimulation (COS) could result in a greater number of overall and metaphase II (MII) oocytes [17,26,27,28], higher fertilization rates [17,26,29], and a greater number of overall generated, top-quality, and cryopreserved embryos [17,26,30]. However, some other studies have reported that GH did not affect the number of overall and MII oocytes [31,32,33], improve embryo quality [31,32], or influence clinical pregnancy rates (PRs) [17,27] or LBRs [17,33,34,35]. The above confusion and equivocal results regarding GH cotreatment with COS, could also be due to heterogeneous patients.

### 2.1. The Application of Growth Hormone in Poor Ovarian Responders

Poor ovarian response is observed in about 9–24% of women who undergo ART [36]. PORs usually have higher basal FSH levels, lower anti-Müllerian hormone (AMH) levels, and lower numbers of oocytes that can be retrieved using COS protocols. Thus, poor ovarian response is an important factor in the success of any modality therapy for infertility and is considered to be a challenging condition for ART specialists due to the high cancellation rates and low LBRs.

There have also been substantial randomized controlled trials (RCTs) that have demonstrated that GH application in ART cycles for PORs exerted positive impacts on the early events of pregnancy, including the number of retrieved oocytes, mature oocytes, good quality embryos and miscarriage (Table 1) [17,21,27,34,37]. However, the effects of GH cotreatment on the late events of pregnancy, such as clinical PRs and LBR, remain controversial [17,27,38,39]. As well as the RCTs, some other studies have shown the effects of GH administration in PORs. In 2017, a single-center retrospective analysis of GH supplementation in 400 IVF patients with poor ovarian response (not using the Bologna criteria) demonstrated that 33–45 days of treatment with 1–1.5 IU of GH increased the chances of clinical pregnancy 3.42-fold (95% CI: 1.82 to 6.44; *p* < 0.0005) and LBR 6.16-fold (95% CI 2.83 to 13.39; *p* < 0.0005) during fresh embryo transfer cycles, after adjustments for maternal age, antral follicle count, and transferred embryo quality [40]. In 2018, a retrospective cohort study enrolled 132 poor responders, according to the Bologna criteria [41] (GH group = 61; control group = 71), who received a mild stimulation protocol [19]. Although the clinical outcomes did not demonstrate statistically significant differences between the two groups due to the limited sample size, there was a trend toward higher rates of clinical PRs (52.4% versus 47.1%; *p* = 0.609) and LBRs (35.7% versus 27.5%; *p* = 0.392) in the GH group [19]. In that study, 4.5 IU of GH was administered nine times (once every 2 days after Day 16 of the previous cycle) and the pregnancy outcomes of the frozen embryo transfer cycles were included in the data analysis. In 2019, a self-controlled clinical study reported that 6 weeks of GH pretreatment using conventional COS protocols (agonist protolcol or antagonist protolcol) increased LBRs (23.5% versus 3.9%; *p* < 0.05) in 380 PORs who had undergone IVF/ICSI fresh embryo transfer cycles. Daily injections of 2 IU of GH were initiated during the previous menstruation cycle until ovum pickup [20].

The controversy surrounding IVF/ICSI cycle outcomes in females could be due to the small sample sizes in studies or differences in the participants inclusion criteria and variable criteria that are used to define PORs. The definition of PORs has varied over time. The RCTs that are listed in Table 1 all defined PORs differently, and most patients were enrolled based on the Bologna criteria [41]. However, a major criticism of the Bologna criteria has been that they represent heterogeneous groups of patients with diverse prognoses. A retrospective cohort study demonstrated that 72% of 225 females with fewer than four oocytes retrieved in the first stimulation cycle were normal responders in a subsequent cycles and became pregnant during the second or third cycle [42]. These results suggested that the criteria could be too broad and that diverse subgroups could actually be created with considerably different prognostic classifications. Additionally, the results from some of the RCTs were not convincing. The RCT that was published by Norman et al. was unable to show any positive effects of GH cotreatment on LBRs in PORs, which could be due to the fact that only 130 cases were recruited in the study (much lower than the 390 planned cases) [43]. Thus, further large-scale RCTs should be performed to explore the application of GH adjuvant therapy for the treatment of females with poor ovarian response.

It is clear that the POR population remains heterogeneous due to the Bologna criteria, primarily because the criteria dose not adequately take the age-related impacts on oocyte quality into consideration. In 2016, the new POSEIDON (Patient-Oriented Strategies Encompassing Individualized Oocyte Number) classification was developed to provide a more detailed classification and reduce the heterogeneity of the Bologna criteria [44]. In order to explore which subgroups of PORs could actually benefit from the GH adjuvant therapy, several studies have recently further defined the subgroups of PORs. In 2019, a retrospective study recruited poor ovarian reserve patients (AMH < 1.2 ng/mL), who were further divided to two groups (a < 35 years of age group and a ≥ 35 years of age group), based on the POSEIDON criteria, and found that GH supplementation using antagonist/agonist protocols greatly improved LBRs (29.89% versus 17.65%; *p* = 0.028) in poor ovarian reserve patients who were older than 35 years old [39]. In 2021, another retrospective study found that GH supplementation improved LBRs (47.66%, 28.33%, 45.45%, and 24.07% in groups 1, 2, 3, and 4, respectively) and clinical PRs (OR: 19.16, 95% CI: 7.87–46.63, and *p* < 0.001 for group 1; OR: 7.44, 95%, CI: 1.65–33.55, and *p* = 0.009 for group 2; OR: 10.19, 95% CI: 2.39–43.52, and *p* = 0.002 for group 3; OR: 27.63, 95% CI: 4.46–171.11, and *p* < 0.001 for group 4) in all four POSEIDON groups [45]. However, in 2020, a retrospective study enrolled 3080 patients who were further divided into POSEIDON group 3 (age < 35 years, AFC < 5, and/or AMH < 1.2 ng/mL) and POSEIDON group 4 (age ≥ 35 years, AFC < 5, and/or AMH < 1.2 ng/mL), and showed that GH adjuvant therapy did not increase LBRs in PORs [46].

Some systematic reviews and meta-analyses, including RCTs and single-center retrospective studies, have concluded that GH administration during ART cycles increased the probability of pregnancy and live births in PORs [2,47,48,49]. These meta-analyses have also found that GH administration appeared to significantly increase LBRs at an odds ratio (OR) of 3.15 (95% CI: 1.26–7.85) [2], and 5.22 (95% CI:1.09–24.99) [49], and increase clinical PRs at an OR of 1.75 (95% CI: 1.23–2.50) [47]. In 2010, a Cochrane review found that the use of GH in PORs produced a significant improvement in LBRs (OR: 5.39; 95% CI: 1.89–15.35), but this study included a small number of RCTs and a small sample size, and it was also unable to identify which subgroup of PORs benefited the most from GH adjuvant therapy [48]. In 2017, another meta-analysis of 11 RCTs that involved 663 females suggested that GH supplementation significantly improved clinical PRs, LBRs, and the number of collected oocytes, while decreasing cancelled cycles rates and gonadotropin doses [18]. In 2020, a meta-analysis of 15 RCTs that involved 1448 patients reported that GH supplementation improved LBRs (RR: 1.74; 95% CI: 1.19–2.54) and clinical PRs (RR: 1.65; 95% CI: 1.31–2.08) [50].

However, some systematic reviews have not reached the same conclusions. In 2020, a meta-analysis of 12 RCTs, which included 536 women who received GH treatment and 553 women who were in a control group, found that GH supplementation did not increased LBRs or ongoing PRs in PORs [51]. Another meta-analysis, which included three studies and a presentation of the Australian LIGHT study, failed to find significant differences in LBRs after GH adjuvant therapy in PORs [52]. One systematic review and network meta-analysis compared the effectiveness of various adjuvant treatment options for POR patients. There were 46 trials that reported on the 6312 women were in this systematic review, and 19 of the 46 trials defined PORs using the Bologna criteria, so 2677 women were included in the network meta-analysis. This meta-analysis found that GH increased the number of retrieved oocytes and lowered the required dosages of gonadotropins for ovarian stimulation, but it did not find that GH adjuvant treatment significnatly improved PRs in PORs [3]. To make the data more homogeneous and easier to compare, this meta-analysis strictly included only 19 RCTs that were based on the Bologna criteria. Thus, the reduced number of RCTs limited the application of the evidence assessment approach to evaluate the overall quality of the evidence, as partial outcomes could not be supported by direct comparative evidence, which affected the final evaluation of results.

The discrepancies among the meta-analyses could be due to the following reasons. Firstly, the definition of PORs varied among the studies that were included in many of the meta-analyses. Since the POSEIDON criteria were developed in 2016, only a few clinical studies enrolled females based on them, and the pre-existing meta-analyses did not include such studies. Secondly, some of the studies that were chosen did not have data for all outcomes, and some lacked data for some outcomes. Additionally, the potential for publication bias regarding the outcome of clinical PRs could not be excluded, which could affect the results. Thirdly, there was no consensus on the dosages of GH for treatment, and the reported dosages ranged from 0.5 IU every other day to 12 IU daily. Fourthly, the proposed ovarian stimulation protocols also presented marked variations in terms of the timing of initiation, dosages, the duration of ovarian stimulation, and the GnRH analog protocols that were used. Finally, the pregnancy outcomes, including cryopreserved embryos and fresh embryos, varied among the included studies, which made it difficult to determine the effects of the heterogeneity of the cases. Therefore, more detailed research application of GH that includes large sample sizes and uses the POSEIDON criteria is needed to better understand the auxiliary role of GH in PORs.

### 2.2. The Application of Growth Hormone in Women with Polycystic Ovary Syndrome

Polycystic ovary syndrome (PCOS) is a common disorder that affects 5–20% of women of reproductive age worldwide [56]. Females with PCOS are inclined to suffer from disruptions of oocyte maturation, follicular development, fertilization, and potential embryonic development competence [57,58], which may negatively influence the outcomes of ART. Though the GH/IGF1 axis plays a role in the pathogenesis of PCOS [59,60], a study on women with PCOS that was resistant to ovulation induction did not derive any benefits from GH supplementation [22]. Recently, one RCT recruited 109 females with PCOS and 50 infertile women who did not have PCOS, and found that GH elevated the fertilization rates of oocytes and the number of cleavage stage embryos [23]. Additionally, GH was proven to improve the rates of implantation and clinical pregnancy, but not significantly [23]. There have been limited studies on the effects of GH cotreatment on clinical pregnancy outcomes for females with PCOS who have undergone ART treatment; thus, more studies, especially RCTs, should be conducted.

### 2.3. The Application of Growth Hormone in Women with Poor Embryonic Development

Early embryonic development is partially affected by maternally derived information, including mRNAs, proteins, and mitochondria [24]. This maternal information is essential for oocyte recruitment, oocytes maturation, fertilization, embryonic development, implantation, and live birth [61,62,63]. Additionally, increasing amounts of evidence have shown that a strong association exists between embryo quality and PR [64,65]. Therefore, successful outcomes of ART are largely dependent on the quality of the retrieved oocytes and embryos during the treatment cycle [66]. However, a limited number of randomized studies have focused on the application of GH in women with poor embryonic development, perhaps due to the difficulties in defining “poor oocytes” or “poor embryonic development”.

One RCT that included 158 patients with poor embryo development has found that GH supplementation increased the number of live births in IVF from July 2017 to February 2019 [24]. Moreover, the cumulus granulosa cells (GCs) from women in the GH group had significantly higher mitochondrial DNA (mtDNA) copy numbers than the cumulus GCs from women in the control group, which indicated that GH administration could benefit the developmental competence of oocytes [24]. GH has also been demonstrated to increase the oocyte cleavage rates in all three age groups (<35 years, 35–40 years, and ≥40 years), according to a cohort study that included 2647 patients with unexplained poor embryonic development during previous IVF procedures [25]. Interestingly, the clinical PRs in the GH group were only found to be significantly higher than those in the non-GH group among females who were more than 40 years of age [25]. Therefore, patients who are more than 40 years of age could benefit more from GH supplementation during IVF cycles than patients who are less than 40 years of age.

### 2.4. The Application of Growth Hormone in Normal Ovarian Responders during ART Treatment

The routine use of GH adjuvant therapy during IVF for normal responders has previously been shown to be of no benefit to ovarian response, embryo quality, or PR [67]. Moreover, recently, a retrospective cohort study that enrolled 41 women, who obtained lower than expected numbers of MII oocytes, poor blastulation rates, and/or lower than expected numbers of euploid embryos for their age in their first cycle of IVF/PGT-A, found that GH supplementation in these women was beneficial and was associated with an increased number of blastocysts for biopsy and a greater number of euploid embryos for transfer [68].

## 3. Molecular Mechanisms of Growth Hormone in Ovarian Functions

Based on the above evidence, we can draw the conclusion that GH appears to be beneficial for the early events of pregnancy when used as an adjuvant therapy during ART cycles [17,26,29]. However, it is still disputed whether GH cotreatment can improve the late events of pregnancy (PRs and LBRs). In addition to more powerful extensive studies, the molecular mechanisms of GH that are involved in ovarian functions (e.g., ovarian follicular growth [4], the activation of primordial follicles [69,70,71,72], steroidogenesis, oocyte quality, etc.) also need our attention.

### 3.1. Effects of Growth Hormone on the Regulation of Primordial Follicles

**Figure 1:**
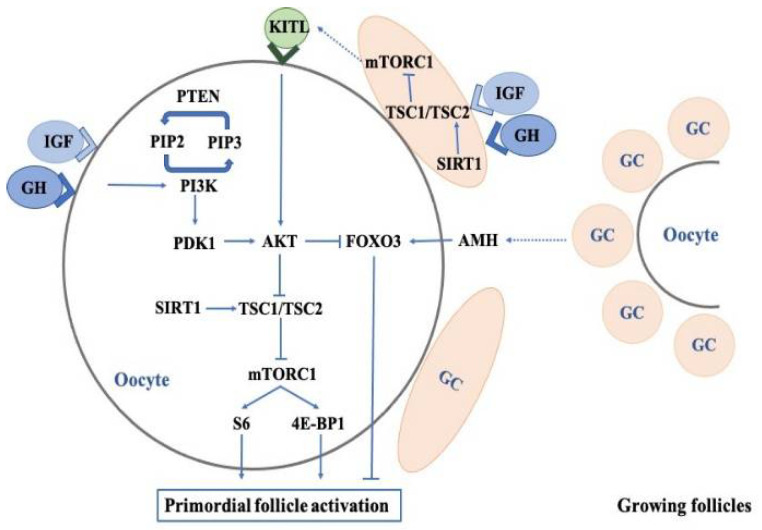
The pathways and their roles in the regulation of primordial follicle activation and quiescence.

According to the follicle developmental stage and gonadotropin dependence, their development can be classified into the following three phases: gonadotropin-independent phase, gonadotropin-responsive phase, and gonadotropin-dependent phase. After birth, primordial follicles are the major follicles in the ovaries, and then some of them begin to grow into primary follicles. This process is called the activation of primordial follicles, which is associated with the following pathways: (a) the activation of the phosphatidylinositol 3-kinase (PI3K)-phosphatase and tensin homolog (PTEN)-protein kinase B (AKT)-forkhead box O3 (FOX3) signaling pathway [69,70]; (b) the activation of the mammalian target of the rapamycin (mTOR) signaling pathway; and (c) the inhibition of AMH [71,72]. The deregulation of these signaling pathways in oocytes contributes to pathological conditions, including premature ovarian failure (POF) and infertility. Accumulated studies have demonstrated that the effects of GH/IGF on primordial follicle activation could be mediated by the PI3K-PTEN-AKT-FOX3 and mTOR signaling pathways and AMH in GCs and oocytes (Figure 1).

Multiple pathways, including PI3K-PTEN-AKT-FOXO3, mTOR-KITL, GH/IGF1, AMH, and SIRT, converge in the mammalian ovaries to regulate the quiescence and activation of primordial follicles.

The abbreviations used are as follows: GH, growth hormone; IGF1, insulin-like growth factor 1; GC, granulosa cell; KITL, KIT ligand; PIP2, phopsphatidylinositol bisphosphate; PIP3, phosphatidylinositol triphosphate; PTEN, phosphatase and tensin homolog; PDK1, phosphoinositide-dependent kinase-1; AKT, protein kinase B; FOXO3, forkhead box O3; PI3K, phosphatidylinositol 3 kinase; AMH, anti-Müllerian hormone; SIRT1, sirtuin 1; TSC1, tuberous sclerosis complex 1; TSC2, tuberin; mTORC1, mTOR complex 1; S6, ribosomal protein S6; 4E-BP1, eukaryotic initiation factor 4E-binding protein 1.

Young female GHR knockout mice have shown increased numbers of primordial follicles and lower serum IGF1, but also harbored a decreased number of follicles in the antral and preovulatory stages [4,73,74], which suggested a slower rate of primordial follicle activation in the above mice. In addition, Saccon et al. also confirmed similar results in GH-deficient Ames Dwarf mice (df/df mice), and further found that df/df mice had fewer GCs surrounding oocytes than normal/df mice in both primordial and primary follicles, and as well as smaller oocyte nuclei diameters in the primordial follicles and oocyte diameters in secondary follicles [75,76]. The oocytes in the primordial and primary follicles of overexpressed bovine GH mice had increased nuclei and oocyte diameters [76]. GH treatment enhanced the progression of primordial follicles into primary follicles, and promoted follicle growth and viability in mouse and goat follicles that were cultured in vitro [77,78].

#### 3.1.1. PI3K-PTEN-AKT-FOXO3 Signaling

The PI3K-AKT pathway is an intracellular signal transduction system that regulates numerous aspects of cell functions, including cell cycle progression and arrest, tumorigenesis, proliferation, and apoptosis [79]. Addtionally, the PI3K-AKT pathway is considered to be involved in both in vivo and in vitro primordial follicle activation [80,81,82,83]. Phosphatase and tensin homolog (PTEN) is a major negative regulator of PI3K and acts to suppress the PI3K-AKT pathway, which can help to maintain the quiescence of the primordial follicles in ovaries for a long time [84]. When the inhibition of PTEN is removed, specifically in the oocytes, FOXO3, which is a downstream factor of the PI3K-PTEN-AKT pathway, is phosphorylated and exported from the nucleus and degraded in the cytoplasm, which then initializes primordial follicle activation [82,85]. Substantial studies have demonstrated that the quiescent state of primordial follicles was associated with the non-phosphorylated form of FOXO3a [81]. Once FOXO3a is phosphorylated, the primordial follicles are activated and begin to grow [85]. Female Foxo3a knockout mice have shown a phenotype of global follicular activation that resulted in oocyte death and the early depletion of functional ovarian follicles due to the global follicular activation [86].

Some publications have proved that the effects of GH/IGF on the primordial follicle activation were associated with PI3K-PTEN-AKT-FOXO3 signaling. Increased numbers of activated follicles were found in the ovine ovarian fragments that were treated with 100 ng/mL of IGF1 and the activation that was caused by the IGF1 was suppressed by the PI3K inhibitor LY294002, which suggested that IGF1 promoted the activation of primordial follicles [87]. An in vitro study further showed that IGF1 reduced pAKT expression in follicles. Additionally, the effects of GH/IGF1 have been demonstrated to be mediated by FOXO3a, as the oocytes in the primordial/primary follicles of GH-deficient mice had lower levels of phosphorylated FOXO3a [76,85]. Consistent with these findings, phosphorylated FOXO3a levels were higher in mice that were treated with GH and in overexpressed bovine GH mice [76]. Thus, the primordial follicle activation that is caused by GH/IGF1 could be initiated by the activation of the PI3K-PTEN-AKT-FOXO3 pathway [87].

#### 3.1.2. The mTOR Pathway

The mammalian target of rapamycin complex 1 (mTORC1)-KITL cascade in primordial follicle GCs and the KIT-PI3K signaling in oocytes are involved in the primordial follicle activation [88]. Additionally, increasing amounts of evidence have suggested the existence of interaction between the mTORC1 pathway and the AKT pathway. mTORC1 is another downstream substrate of AKT, which is further phosphorylated by the mammalian target of rapamycin complex 2 (mTORC2) at serine 473 for its full activation, and then regulates follicle development, including primordial follicle activation [80]. Tuberous sclerosis complex 1 (TSC1) and TSC2, which are two heterodimeric complexes, negatively regulate mTORC1. *Tsc1* knockout and *Tsc2* knockout mice have shown the premature activation of all primordial follicles in the mouse oocytes, which indicated that the over-activation of mTORC1 signaling promoted follicular activation [89,90]. Consistent with these findings, Fmr1 knockout mice were shown to be at a higher risk of POI with increased levels of mTOR, which could be alleviated by the utilization of the mTOR inhibitor rapamycin [91]. Treatment with mTOR stimulators (phosphatidic acid and propranolol) has been proven to accelerate primordial follicle activation in human ovarian cortex cubes in vitro [83]. Additionally, cap-dependent translation initiation in Tsc1^−/−^ oocytes has been enhanced by the increased phosphorylation of rpS6 and eIF4B, which are two downstream effectors of mTORC1 signaling [90]. Therefore, the above studies demonstrated that the activation and inhibition of mTOR signaling were associated with the activation and quiescence of primordial follicles, respectively.

GH-deficient and GH-resistant mice have shown many phenotypic characteristics that presumably contributed to healthy aging, extended longevity, and delayed maturation [92,93]. Reduced mTORC1 and increased mTORC2 signaling have also been reported in the liver, muscle, heart, and kidney tissue of the GH-deficient and GH-resistant mice [94]. No significant alterations to *mTor* expression have been found in the activated primordial follicles of GH-deficient adult or aged mice, compared to those of normal mice [85]. A tendency toward the higher expression of *mTor* has been reported in the ovaries of GHR knockout mice that were treated with phorbol 12-myristate 13-acetate, which is an inhibitor of GH and IRS1 signaling [95]. Additionally, an in vitro study found that 100 ng/mL of IGF1 could decrease SIRT1 accumulation in porcine ovarian GCs [96]. The overexpression of ovarian SIRT1 could suppress the levels of mTOR in transgenic mice [97]. Conversely, SIRT1 and SIRT3 deficiencies have been shown to accelerate the loss of the primordial follicles and increase mTOR signaling [98]. Taken together, the primordial follicle activation that is caused by GH/IGF1 may be instigated by the activation of the mTOR pathway in the ovaries, but whether and how GH/IGF1 activates primordial follicles via the regulation of primordial GCs and oocytes remains unclear.

#### 3.1.3. Anti-Mullerian Hormone

AMH is a growth factor that belongs to the transforming growth factor-β (TGF-β) superfamily. In females, AMH is secreted by the GCs in preantral follicles and small antral follicles (~6 mm in diameter) and is believed to be an excellent marker for the ovarian follicle pool and, indirectly, the primordial follicle pool [99]. In AMH knockout mice, primordial follicles have been reported to be globally activated and then depleted, which suggested that AMH maintained the quiescence of primordial follicles [72,100].

The primordial follicle reserves in GH-deficient dwarf mice were greater than those in their normal littermates. Meanwhile, AMH concentrations were seven times higher in GH-deficient dwarf mice than in their normal siblings [101]. However, two cross-sectional studies investigated that the effects of GH treatment on Turner Syndrome (TS) patients during childhood and found a higher frequency of measurable AMH in TS females after GH therapy [102,103]. Additionally, Hamza et al. showed that there were significant positive correlations between AMH levels and GH dosages [102]. However, the following reports found that GH did not directly affect AMH levels in humans and animals. There were no significant differences in serum AMH levels in TS patients between the GH group and the control group [103]. Similarly, in short prepubescent girls, who were born small for their gestational age, serum AMH levels were also not affected by GH treatment and were similar to those observed in the control girls [104]. Serum AMH levels were not significantly altered in young or aged mice after 8 weeks of injections of recombinant human GH (rhGH) [105]. Thus, these data about the effects of GH on the primordial follicle activation via AMH were not consistent and further studies should be conducted.

### 3.2. Effects of Growth Hormone on Oocyte Quality

Oocyte quality impacts early embryonic survival, as well as the establishment and maintenance of pregnancy and fetal development; thus, it is also regarded as a factor for developmental competence. During folliculogenesis, the oocytes achieve developmental competence. In human ovaries, GHR has been detected in the membranes of cumulus cells [106] and in the nuclei of mature oocytes [13], which confirmed that GH acted at this level to improve the nuclear and cytoplasmic maturation of mature oocytes, and the expansion of cumulus cells as well [12,107,108]. Additionally, high GH concentrations in human follicular fluid has been reported to be associated with the high developmental competence of oocytes [109]. However, the underlying mechanisms of GH that affect oocyte quality are not entirely known.

#### 3.2.1. Nuclear Maturation

Accumulative RCTs have revealed that GH treatment could also improve the outcomes of assisted conception by increasing the numbers of MII stage oocytes, two-pronuclear zygotes (2PN), and transferred embryos [17,33,37,55,110]. The in vivo administration of GH has also been reported to promote the in vitro maturation (IVM) of the human germinal vesicles of human oocytes that were retrieved from small antral follicles [111]. The maturation rates of equine [11,107,112], mouse [113], and human oocytes [108,114] have been demonstrated to be improved by GH. Recently, Lin et al. reported that the proportion of morphologically abnormal spindles in GH-treated mouse oocytes was significantly lower than that in the control oocytes [115] Additionally, single-cell RNA-Seq and real-time PCR data have shown that GH could significantly enhance the expressions of aurora kinase A (*AURKA*) and centromere protein E (*CENPE*) [108]. AURKA localizes at the meiotic spindle poles and contractile rings/midbodies during the first polar body extrusion and plays a role in microtubule assembly and spindle organization [116,117]. AURKA is also involved in the germinal vesicle breakdown and meiotic resumption [117,118,119]. As an essential meiotic kinetochore motor, CENPE is required for meiotic progression. Most mouse oocytes have been found to be arrested at the MI stage and fail to extrude the first polar body after a microinjection of the CENPE antibody [120]. Furthermore, the exogenous GH treatment has been found to reduce the proportion of abnormal spindles, possibly by promoting cytoplasmic maturation, including mitochondrial functions, which has been reported to support the spindle assembly [115].

#### 3.2.2. Cytoplasmic Maturation

While acquiring developmental competence, oocyte cytoplasm matures with an accumulation of mRNA, proteins, substrates, and nutrients, which ultimately fosters embryonic developmental competence. The distribution of cortical granules in the cortical cytoplasm is specific to immature oocytes while the distribution of cortical granules just beneath the plasma membrane is specific to mature oocytes [121,122], and thus the cortical granule distribution has been used as a reliable indicator for the cytoplasmic maturation of oocytes. The addition of GH/IGF1 to an in vitro maturation (IVM) medium has influenced the in vitro cytoplasmic maturation of equine oocytes, which was required to achieve complete maturation competence [123,124]. However, how GH influences the migration of cortical granules in oocytes is poorly understood.

As well as the migration of cortical granules, cytoplasmic maturation can also be evaluated using some other parameters, including the distribution and functions of mitochondria and intra-oocyte adenosine triphosphate (ATP) levels. During oocyte maturation, there is extensive mitochondrial redistribution, and high ATP levels are required to meet the high-energy demands of oocyte processes, including spindle formation, chromosomal segregation and meiotic division, fertilization, and embryonic division [125,126]. Lin et al. reported that GH could elevate the mitochondrial membrane potential and ATP concentrations in mouse oocytes without any significant alterations to mitochondrial distributions [115]. Another study also obtained similar results, but further found that GH could increase the frequencies of homogeneous mitochondrial distributions in mouse oocytes [105]. Interestingly, rhGH administration in both aged and young mice has not been found to significantly influence mtDNA copy numbers per oocyte compared to saline administration [105]. Similarly, rhGH has been shown to modulate the energy metabolism of oocytes in terms of mitochondrial membrane potential and ATP content in cyclophosphamide-induced POI rats but not mtDNA copy numbers [127]. In PORs, the administration of GH as an adjuvant therapy to standard ovarian stimulation, significantly increased GHRs, as well as the functions and activity of oocyte mitochondria [128]. GH has been demonstrated to activate various proteins in the β-oxidation or tricarboxylic acid cycles, as well as other components of the mitochondrial fuel delivery and oxidative machinery, thereby enhancing the capacity of oxidative ATP generation [129]. Reduced ATP production, which is caused by mitochondrial dysfunction, is involved in meiotic chromosomal nondisjunction and may limit cell division and embryonic development [130]. Additionally, bone morphogenetic protein 15 (BMP15), which is synthesized in and secreted from oocytes, is found in all cytoplasmic regions of matured oocytes in vitro [131], and plays a pivotal role in stimulating cumulus cell expansion [132] and mediating ATP synthesis in oocytes [133]. Indeed, the expression of *Bmp15* was higher in the ovaries of GHR knockout mice than those of normal mice [95]. Meanwhile, based on single-cell full-length mRNA sequencing technology, a GO analysis and the KEGG pathways for the differently expressed genes (DEGs) revealed that rhGH intervention in cyclophosphamide-induced POI rats was associated with cellular oxidant detoxification and response to peptide hormones within the biological process category [127]. Additionally, the mitochondrion was the main cellular component of these DEGs. As expected, glutathione metabolism and oxidative phosphorylation pathways have been reported to be enriched after rhGH therapy [127]. Thus, the beneficial effects of rhGH on mitochondria could be due to the alleviation of oxidative stress and the detoxification of cellular oxidants.

Taken together, GH could improve the maturation process of oocytes in humans and other species, probably by accelerating meiotic progression, balancing the redox homeostasis of cellular environment, and promoting oocyte developmental competence.

### 3.3. Effects of Growth Hormone on the Sensitivity of Oocytes to Gonadotropins

In some studies, GH supplementation has been shown to reduce the need for gonadotropin and shorten the stimulation time, which suggests that supplementary GH could be capable of increasing the sensitivity of oocytes to gonadotropins [34,55,134]. Indeed, GHR/GH-binding protein knockout mice have been reported to have a significantly lower responsiveness to exogenous gonadotropin treatment [4,74]. Additionally, in vivo and ex vivo studies have revealed that GH supplementation as a part of IVF treatment upregulated the expressions of LHR, FSHR, and GHR in human GCs, when isolated after egg collection from women with decreased ovarian reserves [128,135]. FSHR and LHR signaling in GCs is required for follicular selection and dominant follicle formation. Interestingly, GH also acts to support the maturation process of luteinization by increasing LHR density and by reducing FSHR expression prior to ovulation [135]. The stimulation of protein kinase A (PKA) by GH and the prevention of GH-induced effects by PKA blockers have been reported to suggest that both the stimulatory and inhibitory effects of GH on porcine ovarian cells were probably mediated by the cAMP/PKA system. Based on the above evidence, the beneficial effect of GH on gonadotropin responses are probably via the cAMP/PKA pathway. Interestingly, IGF treatment has not been able to improve either fertility or ovarian responsiveness to exogenous gonadotropins [4]; however, the administration of GH has been experimentally shown to enhance the development of small antral follicles into the gonadotropin-dependent phase in vivo [136]. Thus, the effects of GH on the gonadotropin responses via the regulations of FSHR, LHR, and GHR, as well as the activation of the cAMP/PKA pathway, may be independent from IGF1.

### 3.4. Effects of Growth Hormone on Granulosa Cells and Thecal Cells

#### 3.4.1. Steroidogenesis

**Figure 2:**
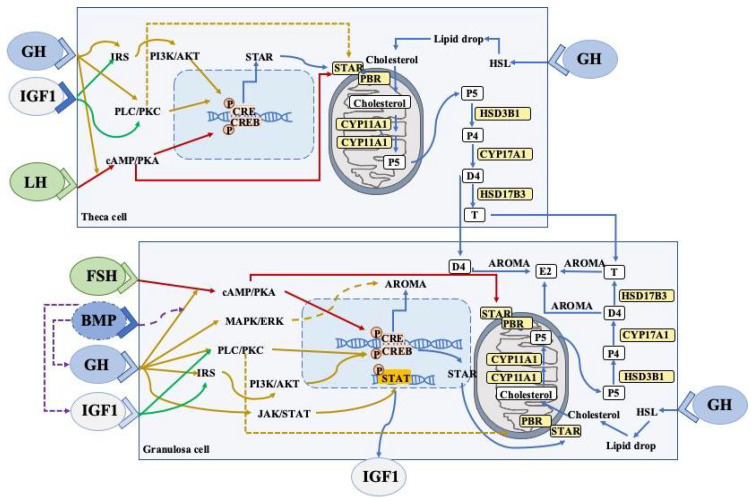
A schematic representation of the major GH/IGF signaling and BMP system networks in ovarian cells (theca and granulosa cells).

During ovarian steroidogenesis, cholesterol is transferred from outer mitochondrial membranes to the inner mitochondrial membranes with the help of an acute regulatory protein (StAR), and is then converted into pregnenolone by CYP11A1, which is a rate-limiting enzyme [137]. Subsequently, CYP17A1 and HSD3B1 sequentially catalyze pregnenolone into androstenedione. Finally, estradiol is generated by the sequential catalyzation of CYP19A1 and HSD17B7 in the GCs [138]. This steroidogenic process can reflect the follicular development and the functions of GCs and theca cells. GH has been demonstrated to affect steroid productions in ovaries, and various intracellular signaling has been shown to be involved in the above process (Figure 2).

GH can induce local IGF1 expression in granulosa cells via JAK-STAT signaling. Both GH and IGF can participate in steroidogenic events and promote cell proliferation via the stimulation of the PLC-PKC and PI3K-AKT pathways, which interact with FSHR and LHR. The expression of aromatase (granulosa cells) and StAR (theca/granulosa cells) is dependent on the CREB pathway. Bone morphogenetic proteins (BMP) suppresses GHR, IGF1, and IGF1R expression, whereas the GH/IGF1 axis downregulates the BMP receptors in the ovaries. Cell proliferation can also be promoted by estrogen and testosterone via autocrine mechanisms.

The abbreviations used are as follows: LHR, luteinising hormone receptor; FSHR, follicle stimulating hormone receptor; cAMP, cyclic AMP; PKA, protein kinase A; CRE, cAMP response element; CREB, cAMP response element binding protein; PLC, phospholipase C, IRS, insulin receptor substrate; PI3K-AKT, phosphoinositide 3-kinase/protein kinase B; StAR, steroidogenic acute regulatory protein; P4, progesterone; P5, pregnenolone; E2, estradiol; D4, androstenedione; T, testosterone; STAT, signal transducer and activator of transcription; AROMA, aromatase, HSL, hormone-sensitive lipase. Arrow, stimulation; Dotted arrows, inhibition.

By binding to the GHRs in the GCs and thecal cells of Graafian follicles, GH has been shown to augment the proliferation of GCs and thecal cells, and to promote steroidogenesis as well [1,139]. Increasing numbers of in vitro studies have found that GH supplementation stimulated the synthesis of progesterone in primary GCs cultures, which could be related to the increased expression of CYP11A1 and HSD3B [8,140]. Moreover, the GH-increased progesterone has been shown to disappear when conditioned media were treated with antiserum against GH [8]. However, there have been contradictory reports about the regulation of GH in the FSH-induced estradiol production [8,140]. Many years ago, 24 normal patients who received short-term GH treatment showed increased E2 production, which was significantly higher than the increase that was induced by FSH injections without GH administration [141]. In isolated preantral caprine follicles, GH has been shown to increase FSH-induced estradiol (E2) production, which could be due to the elevated activity or expression of CYP19A1 [142]. However, another in vitro study found that GH suppressed FSH-induced E2 production, as well as causing reductions in aromatase expression [140]. Interestingly, GH alone has been reported to be unable to induce any steroidogenic responses [141], which implied that the GH steroidogenic effects might be dependent on the FSH actions. Additionally, the incubation of GCs with either recombinant chicken GH or conditioned media that predominantly contained a 15 kDa GH isoform has been shown to significantly increase GC proliferation; meanwhile, the knockdown of local ovarian GH with a specific cGH siRNA in hen GC cultures reduced the proliferation rate of GCs [143]. Thus, GH-mediated disruptions to progesterone and E2 production in GCs may be associated with the expressions of steroidogenic enzymes and the proliferation of GCs.

#### 3.4.2. JAK2-Dependent Signaling Pathway

The binding of GH to the GHR dimer results in the activation of the intracellular Janus Kinase 2 (JAK2) pathway, which can phosphorylate itself and the cytoplasmic regions of GHR [144], and then subsequently phosphorylates STAT molecules (particularly STAT5a, 5b, 1, and 3). STAT5b is considered to be of the utmost importance as it directly regulates the expression of IGF1 [145] and has been shown to act as a mediator of GH-induced IGF1 production in rat GCs [140,146]. When the transcription molecule STAT is recruited, the subsequently mitogen-activated protein kinases (MAPK)/phosphatidylinositol 3-kinase (PI3K)/protein kinase C (PKC)/phospholipase A2 (PLA2) pathway is stimulated. MAPK activation has been demonstrated to regulate FSH-mediated steroidogenesis in the GCs [147,148]. ERK1/2 is involved in the synthesis of progesterone and E2 which is induced by FSH [149]. GH can also enhance FSH-induced ERK signaling, which is likely to be involved in the effects of GH on FSH-induced E2 and progesterone production [140]. Considering that the inhibition of IGF1 blocks the GH-induced stimulation of FSH-induced progesterone production, GH affects the production of steroid hormones via the JAK/STAT and ERK pathways, which are functionally involved in the activation of IGF1 expression. Based on the fact that BMP regulates steroidogenesis and the mitosis of GCs, one study revealed that GH/IGF1 actions impaired BMP-SMA and Mad-related protein 1/5/8 signaling, and that BMP in turn inhibited the IGF1 effects that increased FSH-induced E2 production via the suppression of the expression of the GH/IGF1 system [140]. The effects of GH on the steroidogenesis of GCs have been demonstrated to be enhanced in the presence of the BMP antagonist noggin [140].

#### 3.4.3. JAK2-Independent Signaling Pathway

Other studies have also found that when GH bound to GHR, the PLC/PKC pathway is also activated, which is independent from JAK2 recruitment [146,150]. PKC has been demonstrated to directly trigger cyclic AMP response-element binding protein (CREB)-mediated gene transcription and, subsequently, StAR and/or aromatase expression in GCs, for example [151]. StAR can bind to cholesterol in the cytosol and transport it to outer mitochondrial membranes, where peripheral-type benzodiazepine receptors (PBRs) are involved in its transport from the outer to inner mitochondrial membranes. However, the phosphorylation of StAR by PKC inhibits the transportation of cholesterol in GCs by interfering in StAR-PBR interactions [152].

#### 3.4.4. FSHR Pathway

FSH exerts its actions on GCs mainly through the stimulation of the cAMP-PKA pathway [153,154,155]. CREB, which is a PKA substrate, is known to play an important role in the regulation of the FSH responsive genes (*Star* and *Cyp19a1*) in GCs [155,156]. Additionally, the phosphorylation of StAR by PKA stimulates cholesterol transportation by enhancing the interactions between StAR and PBR [152]. Accumulated evidence has shown that GH supplementation could elevate the expressions of LHR, FSHR, and GHR in human GCs [128,135]. Thus, GH can regulate the steroidogenesis of GCs via the FSHR pathway.

#### 3.4.5. GH/IGFs Signaling Pathway

The GH–GHR interaction can induce the production of ovarian IGF1 via the GHR/JAK2/STAT5b pathway [145]. Several GH-deficient mouse models have shown significantly reduced plasma IGF1 concentrations [157,158]. As well as IGF1, other members of the IGF family have also been shown to be influenced by GH [6]. The IGF system comprises two ligands (IGF1 and IGF2), three receptors (IGF1R, IGF2R, and insulin receptors), six secreted IGF-binding proteins (IGFBPs), and IGFBP proteases [159]. Female IGF1R knockout mice have been shown to have ovaries with no antral follicles and demonstrate a 90% reduction in serum E2 levels [160]. Additionally, IGF1 knockout mice have also been shown to exhibit the decreased expression of FSH receptors and, consequently, reduced aromatase expression and E2 secretion [161]. An in vitro study also confirmed that the inhibition of IGF1 signaling restored GH-induced stimulation of FSH-induced progesterone production, which suggested that endogenous IGF1 could be functionally involved with the effects of GH on progesterone production [140]. The biological effects of IGF1 have been further demonstrated to be mediated by IGF1 binding proteins, which are synthesized and secreted in human ovarian GCs [162]. Interestingly, increased free IGF1 has been demonstrated to decrease the synthesis of IGF2R, thereby allowing for more IGF2 to be bioavailable (free) for the induction of steroidogenesis and mitogenesis via the IGF1R [163]. Thus, the above evidence has suggested that by regulating the sensitivity of GCs to gonadotropin, IGF1 and IGF2 could be important downstream factors of GH in female reproduction and that the physiological effects of GH on ovary functions could be due to the direct and/or indirect action of GH.

### 3.5. Mitochondrial Functions

Oxidative stress is induced by the overproduction of reactive oxygen species (ROS), which are mainly generated by mitochondria [164]. Therefore, mitochondrial dysfunction can lead to pathological changes, disruption to the production of normal ATP levels, and increases in the levels of ROS [165]. Oxidative stress is also considered to be one of the important aspects that causes reproductive dysfunction, including abnormal follicular atresia, ovum meiosis, lower fertilization rates, and delayed embryonic development [166]. Additionally, oxidative stress can initiate during several reproductive diseases, including PCOS [167], endometriosis [168], premature ovarian failure, etc. Mitochondrial dysfunction that is caused by ROS is also involved in reduced oocyte developmental competence and fertilization failure [165]. Recently, GH has been widely applied in the treatment of the above reproductive pathologies, which could be due to its downregulation of oxidative stress in ovaries. GH has been shown to alleviate the total oxidative stress and oxidative stress index level in follicular fluid, and increase GC mitochondrial membrane potential in Chinese patients with PCOS [23]. Additionally, the GCs in the above patients suffered from higher apoptosis, which could be associated with the decreased function of the PI3K-AKT signaling pathway; meanwhile, GH could alleviate caspase-dependent apoptosis of GCs and activate the PI3K-AKT signaling pathway in GCs [169]. Recent studies on mammals have confirmed that the PI3K-AKT signaling pathway could regulate the growth and apoptosis of GCs during follicular development [170,171]. Similarly, GH has been found to reduce ROS-induced apoptosis in some types of cells including the vascular endothelium, cardiomyocytes, and neural and skeletal muscle cells, by activating the PI3K-AKT signaling pathway [172,173]. Furthermore, GH has been shown to exert protective effects on cisplatin-induced ovarian GC apoptosis by downregulating oxidative stress and enhancing mitochondrial functions (i.e., mitochondrial membrane potential and mtDNA copy numbers) via the Sirt3-Sod2 pathway [174]. Sirt3 exists in the mitochondrial matrices and participates in the regulation of mitochondrial functions, as well as acting as an oxidative stress sensor and playing a protective role in controlling ROS generation [175]. In addition to directly reducing the production of ROS, Sirt3 can also regulate the acetylation levels of manganese superoxide dismutase (Sod2), thereby promoting the detoxification of ROS and suppressing mitochondrial oxidative stress [174]. Thus, GH may alleviate apoptosis by lowering the levels of ROS, elevating mitochondrial membrane potential, and recovering mtDNA copy numbers in GCs.

## 4. Conclusions

GH has been used as an adjuvant therapy to ovarian stimulation for about 30 years; however, its place and influence are yet to be clarified. GH is widely used in clinical practice, including PORs, and patients with PCOS and poor embryonic development, and exerts beneficial effects on early pregnant events. GH has also been shown to have multiple effects on female reproductive physiology, such as the activation of primordial follicles, the enhancement of gonadotropin sensitivity, the promotion of steroidogenesis, as well as improving oocyte quality, all of which may be involved in early pregnancy outcomes. Additionally, the above GH-mediated effects are associated with several intracellular pathways (Figure 1 and Figure 2).

Insights into current applications, physiologic effects, and mechanisms of GH could be beneficial for promoting the proper usage of GH in clinical practice. However, the effects of GH on LBRs remain unclear, which could be mainly due to the following reasons: (I) the small sample sizes; (II) heterogeneous patients being enrolled in RCTs; (III) the varying dosages of GH, diverse POR definitions, and different ovarian stimulation protocols (the timing of initiation, the dosages, the duration, and the GnRH analog protocols) that have been included in meta-analysis. Additionally, previous studies have found that GH and IGF1 could promote progesterone synthesis in granulosa cell cultures [8,140,176]; however, elevated progesterone levels on the day of hCG administration negatively influence PR during IVF, regardless of different ovarian responses [177]. Until now, GH has mostly been applied during COS (from the day of gonadotropin administration to the day of hCG administraition), as the elevated serum progesterone that is induced by GH/IGF could affect the late outcomes of pregnancy. Additionally, few studies have focused on long-term treatment with GH during ART, and there have been no reported beneficial effects of using GH during ART due to insufficient periods of GH administration. Thus, the therapeutic windows and duration of GH administration during ART should be further investigated in the future.

## Figures and Tables

**Table 1:** The randomized controlled studies on growth hormone cotreatment in poor ovarian responders.

ART (Year)	Inclusion Criteria	Main Exclusion Criteria	GH Usage /Stimulation Protocol	Clinical Outcomes
IVF (2018)	127 females (according to the Bologna consensus criteria [41])	BMI > 30 mg/m^2^, and women with other causes of infertility	Sustained-release of 20 mg of GH three times before and during COS/GnRH-Ant protocol	GH elevated the follicles number and the proportion of metaphase II oocytes; there was no significant differences in the percentages of clinical and ongoing pregnancy, or miscarriage [21].
IVF (2019)	184 females (according to the Bologna consensus criteria [41])	No description	Injection of GH at dosages of 4, 4, and 2 IU over three successive days, along with the ovulation induction/GnRH-a ultra-long protocol	GH elevated the ovarian response, the number of retrieved oocytes, the number of embryos available for transfer, clinical pregnancy rates, and ongoing pregnancy rates; there were no significant differences between groups regarding miscarriage rates [53].
IVF/ ICSI (2019)	130 females (≤1 IVF cycle, with ≤5 oocytes, rFSH dosage > 250 IU/d, basal FSH ≤ 15 IU, BMI < 33 kg/m^2^, and age < 41 years)	Females with a history of malignant diseases or pituitary/hypothalamic diseases; current ovarian cysts (>3 cm) or chronic infectious diseases, PCOS, or AUB	Daily injection of 12 IU GH from day 1 of COS until the day of hCG/GnRH-Ant protocol	GH increased the number of retrieved oocytes and did not signficantly affect the LBRs or the number of transferred embryos [43].
IVF/ ICSI (2019)	105 females (according to the Bologna consensus criteria [41])	FSH > 20 IU/L, a history of infertility due to non-POR causes	Daily subcutaneous injection of 2.5mg of GH from the eighth day of the cycle until the injection of HCG /GnRH-Ant protocol	GH significantly increased the number of retrieved oocytes, MII oocytes, fertilized oocytes, transferred embryos, and clinical PRs [54].
IVF/ ICSI (2015)	145 females (according to the Bologna consensus criteria [41])	FSH > 20 IU/L, women with other causes of infertility, and severe male factors	Daily injection of 2.5 mg of GH from day 6 of COS until the day of hCG/GnRH-a long protocol	GH increased the number of retrieved oocytes, MII oocytes, and the mean number of fertilized oocytes and elevated PRs without any significant differences between groups [38].
IVF/ ICSI (2017)	50 females (according to the Bologna consensus criteria [41])	FSH > 20 IU/L, BMI ≥ 35 kg/m^2^, and severe male factors	Daily injection of 4 IU of GH from day 1 of COS until the day of hCG/GnRH-Ant protocol	GH lowered the effective dose of Gn and the duration of stimulation while elevating the total number of oocytes, as well as the numbers of MII oocytes, 2PN zygotes, and good-quality transferred embryos and the probability of pregnancy [37].
IVF/ ICSI (2018)	240 females (according to the Bologna consensus criteria [41])	>45 years, FSH > 20 IU/L, tubal occlusion, and severe male factors	Daily injection of 7.5 IU of GH from day 21 of the previous cycle until the day of hCG/GnRH-a long protocol	GH improved the numbers of retrieved oocytes, MII oocytes, fertilized oocytes, transferred embryos, and cryopreserved embryos; there were no significant differences in the LBRs, whether fresh or cumulative [34].
IVF/ ICSI (2016)	141 females (according to the Bologna consensus criteria [41])	FSH > 20 IU/L, and women with other causes of infertility	Daily injection of 7.5 IU of GH from day 6 of COS until the day of hCG/GnRH-Ant protocol	GH lowered the effective dose of Gn and the duration of GnRH-Ant treatment while increasing the numbers of collected oocytes, MII oocytes, fertilized oocytes, and transferred embryos, and as well as the mean E2 levels on the day of hCG; there were no significant differences in clinical PRs per cycle or LBRs per cycle [17].
IVF/ ICSI (2013)	82 females (≥1 previous failed IVF-ET cycles with ≤3 retrieved oocytes and ≤3 subsequently obtained embryos using GnRH-a long protocol, and/or E2 levels ≤ 500 pg/mL on the day of hCG)	BMI ≥ 30 mg/m^2^, FSH > 15 IU/L, women with other causes of infertility, and azoospermia	Daily injection of 4 IU of GH from day 21 of the previous cycle until the day of hCG/GnRH-Ant protocol	GH increased the numbers of retrieved oocytes and obtained embryos; there were no significant differences between groups regarding implantation, or chemical and clinical PRs [27].
IVF (2015)	64 females (according to the Bologna consensus criteria [41])	BMI ≥ 30kg/m^2^, women with other causes of infertility, altered karyotype in couples, and severe male factors	Daily injection of 0.5 IU of GH from day 1 of the agonist until the day of hCG/GnRH-a long protocol	GH increased the numbers of top-quality embryos and cryopreserved embryos[28].
ICSI (2008)	61 females who responded poorly to high doses of gonadotropin treatment during their first cycles in the same center	D3 FSH > 20 IU/L	Daily injection of 12 IU of GH from day 21 of the precious cycle until the day of hCG/GnRH-a long protocol	GH increased zygotes; although more pregnancies and more clinical pregnancies with fetal heart beat were achieved in the GH group (12/31), compared to the control group (6/30), the difference was not statistically significant [55].

D2-3, at day 2–3 of the menses; AUB, abnormal uterine bleeding; COS, control ovarian stimulation; MII oocytes, metaphase II stage oocytes; 2PN zygotes, two pronucleus zygotes; BMI, body mass index; GnRH-Ant, gonadotropin-releasing hormone antagonist; GnRH-a, gonadotropin-releasing hormone agonist; LBR, live birth rate; Gn, gonadotropin; PR, pregnancy rate; PCOS, polycystic ovary syndrome; hCG, human chorionic gonadotropin; PGT, preimplantation genetic testing.
